# One-Step Fabrication of Recyclable Konjac Glucomannan-Based Magnetic Nanoparticles for Highly Efficient Cr(VI) Adsorption

**DOI:** 10.3390/molecules28207100

**Published:** 2023-10-15

**Authors:** Jianjuan Zhang, Huiyun Ren, Honglei Fan, Shaofeng Zhou, Jin Huang

**Affiliations:** 1 School of Environment and Safety Engineering, School of Chemistry and Chemical Engineering, School of Materials Science and Engineering, North University of China, Taiyuan 030051, China; 2 Chongqing Key Laboratory of Soft-Matter Material Chemistry and Function Manufacturing, School of Chemistry and Chemical Engineering, Southwest University, Chongqing 400715, China

**Keywords:** one-step precipitation method, konjac glucomannan, magnetic nanoparticles, chromium adsorption, recyclable

## Abstract

Recently, the natural polymer polysaccharide konjac glucomannan (KGM) has received attention as a promising adsorbent in water treatment due to its low toxicity, cost-effectiveness and biocompatibility. However, the high-level water absorbency of KGM makes it difficult to recover in water treatment. In this study, by combining KGM with magnetic nanoparticles, KGM-based magnetic nanoparticles (KGM-Fe_3_O_4_ NPs) with excellent adsorption properties and recyclability for heavy metals were prepared using an one-step precipitation method. The as-prepared KGM-Fe_3_O_4_ NPs have a spherical morphology of superparamagnetism with a small particle size (ca. 7.0 nm) and a large specific surface area (160.1 m^2^·g^−1^). Taking Cr(VI) as the target heavy metal ion, the above nanoparticles have a high adsorption capacity and fast adsorption rate for Cr(VI). The pseudo-second order kinetic model is more suitable to describe the adsorption process of Cr(VI) by KGM-Fe_3_O_4_ NPs, and the maximum adsorption capacity of Cr(VI) onto KGM-Fe_3_O_4_ NPs was calculated to be 41.67 mg·g^−1^ using the Langmuir isotherm model. In addition, KGM-Fe_3_O_4_ NPs with adsorbed heavy metal ions can be quickly recovered from a solution, regenerated, and reused in the next cycle. KGM-based Fe_3_O_4_ nanoparticles are promising adsorbents that show significant reusability for the removal of metal ions in water and wastewater treatment.

## 1. Introduction

With the rapid advance of industrialization, the problem of water pollution, especially heavy metal ions in water, is becoming an increasingly serious and threatening environmental safety and human health [1,2]. Chromium (Cr) contamination, as one of the challenges, is mainly caused by leather tanning, electroplating, metalworking, printing, pigments and chrome mining [3]. Cr(III) and Cr(VI) are the two valence states of chromium present in industrial wastewater, of which, trace Cr(III) is an essential trace element, while Cr(VI) is toxic, carcinogenic and highly water-soluble, and the toxicity of Cr(VI) is about 500 times more than that of Cr(III) [4,5,6]. Therefore, it is very urgent to deal with the problem of Cr(VI) contamination. Until now, a variety of methods have been used to treat Cr(VI) contamination, for example, chemical precipitation [7], ion exchange [8,9], membrane filtration [10], solvent extraction, coagulation and adsorption [11,12]. Among these methods, adsorption technology is more economical than membrane filtration is, easier to operate than coagulation/precipitation is, and more versatile than ion exchange is, which is considered as the most practical technology [13,14,15,16].

As a core component of the adsorption method, sorbents are one of the key issues in environmental science and materials science research. Currently, there are mineral, microbial and biomass adsorbents for the treatment of heavy metal water pollution, but due to the shortage of resources and secondary pollution, there is an urgent need to find new adsorbents [17,18,19,20]. Konjac glucomannan (KGM) is a high-molecular-weight, water-soluble, non-ionic polysaccharide, which isolated from the tubers of Konjac. KGM is a linear random copolymer consisting of D-glucose and D-mannose linked in a molar ratio of 1:1.6 and containing a large number of hydroxyl and acetyl groups in the molecular chain. This KGM has received considerable attention due to its low toxicity, cost-effectiveness and biocompatibility [21]. Something of interest is that KGM-based materials have shown effective adsorption properties with various types of water-soluble contaminants, including heavy metals, polyphenols and polyaryl dyes due to the excellent hydrogen bonding and chelation effects of hydroxyl groups [22,23,24,25]. However, the high-level water absorbency of KGM makes it impossible to use KGM directly as an adsorbent, which requires modification to properly control its water absorption [23]. At present, the modification of KGM adsorbent materials mainly consists of cross-linking and gelation. Crosslinked carboxymethyl konjac glucomannan (CMKGM) was prepared through a reaction with monochloroacetic acid and used to adsorb Pb(II), Cu(II) and Cd(II), which show a high adsorption capacity for Pb(II) with 41.7 mg·g^−1^ [24]. KGM-based magnetic carbon aerogels with excellent arsenite and dyes removal properties were fabricated via a two-step process including the preparation of the magnetic cores and gelation with KGM [25]. Unfortunately, the adsorbents obtained in the above two ways suffer from a low mechanical strength and difficulty in the efficient adsorption of heavy metal ions due to the high-level consumption of hydroxyl groups by the crosslinker.

Recently, magnetic nanoparticles have attracted the intensive attention of many researchers and been widely used in heavy metals in wastewater treatment, owing to their magnetic properties, high surface area, chemical stability, easy synthesis and low toxicity [26,27]. In particular, its magnetic properties allow it to be quickly separated from aqueous solutions, avoiding secondary contamination. In order to increase the adsorption capacity of the KGM adsorbent, magnetic Fe_3_O_4_ nanoparticles were used as a carrier to enable the stable presence of KGM in aqueous solutions. In this present work, we provide a method that enables the rapid and controlled preparation of KGM-based magnetic nanoparticles (KGM-Fe_3_O_4_ NPs) using an one-step precipitation method. The structure and morphology of KGM-Fe_3_O_4_ NPs adsorbents were characterized in detail, and their adsorption of Cr(VI) in aqueous solutions was investigated under different experimental conditions. In addition, the reusability of KGM-Fe_3_O_4_ NPs for Cr(VI) was also investigated.

## 2. Results and Discussion

### 2.1. Characterization of KGM-Fe_3_O_4_ NPs

**Figure 1:**
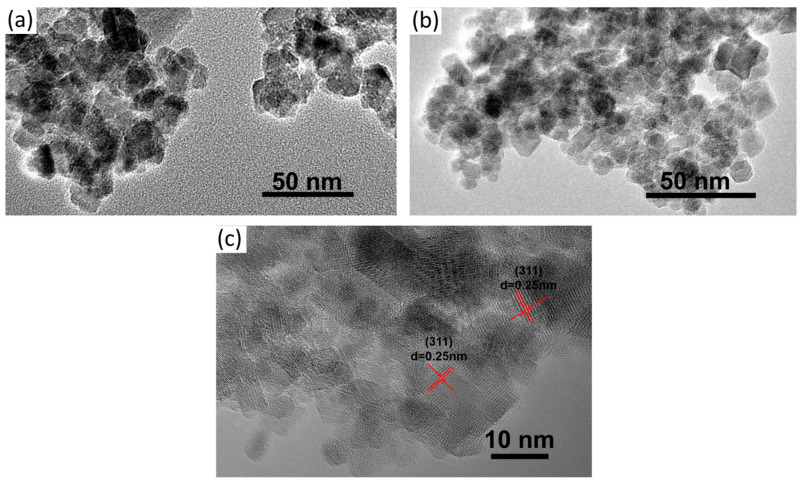
TEM images of (**a**) Fe_3_O_4_, (**b**) KGM-Fe_3_O_4_ NPs and (**c**) HRTEM image of KGM-Fe_3_O_4_ NPs.

**Figure 2:**
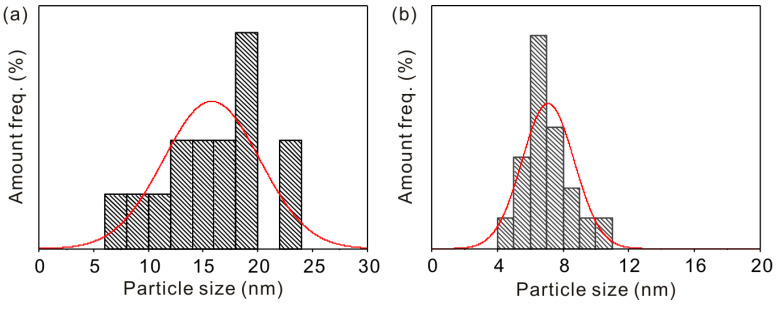
Particle size distribution of (**a**) Fe_3_O_4_ and (**b**) KGM-Fe_3_O_4_ NPs.

The morphology of Fe_3_O_4_ and KGM-Fe_3_O_4_ NPs are shown in Figure 1. As shown in Figure 1a, it can be seen that the Fe_3_O_4_ nanoparticles were sphere-like, with slight aggregation. From Figure 1b, it appears that the morphology of KGM-Fe_3_O_4_ NPs was still quasi-spherical, with improved dispersion compared to that of Fe_3_O_4_. In this research, from the HRTEM image (Figure 1c), a number of (311) planes of the Fe_3_O_4_ nanoparticles with a lattice fringe spacing of 0.25 nm were detected. The TEM particle sizes of Fe_3_O_4_ and KGM-Fe_3_O_4_ NPs were statistically calculated using Nano Measurer software (1.2.0.5), and the results are shown in Figure 2a,b. The average particle size of Fe_3_O_4_ was approximately 16.0 nm, with a particle size distribution in the range of 8–23 nm. While the KGM-Fe_3_O_4_ NPs had smaller particle size (ca. 7.0 nm) and narrower particle size distribution (5.0–11 nm). This is due to the konjac glucomannan chains preventing the nanoparticles from agglomerating in the preparation.

**Figure 3:**
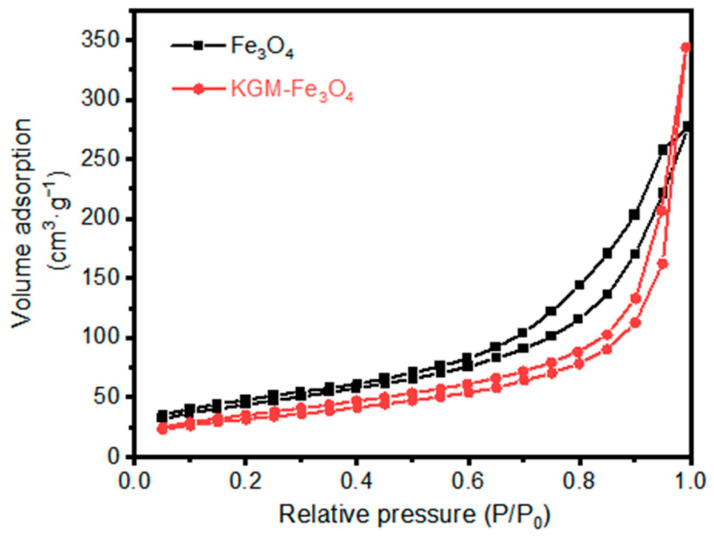
Nitrogen adsorption–desorption isotherm of Fe_3_O_4_ and KGM-Fe_3_O_4_ NPs.

Furthermore, the surface areas of the as-prepared Fe_3_O_4_ and KGM-Fe_3_O_4_ NPs were calculated using N_2_ adsorption–desorption isotherms (Figure 3). As obtained from the N_2_ adsorption–desorption isotherms, the surface areas of the as-prepared the Fe_3_O_4_ and KGM-Fe_3_O_4_ NPs were 140.4 and 160.10 m^2^·g^−1^, respectively. Compared to the Fe_3_O_4_ nanoparticles, the steric hindrance of KGM macromolecular chains can hinder the agglomeration of Fe_3_O_4_ particles and improve the dispersion of Fe_3_O_4_ nanoparticles, thereby increasing their specific surface area.

**Figure 4:**
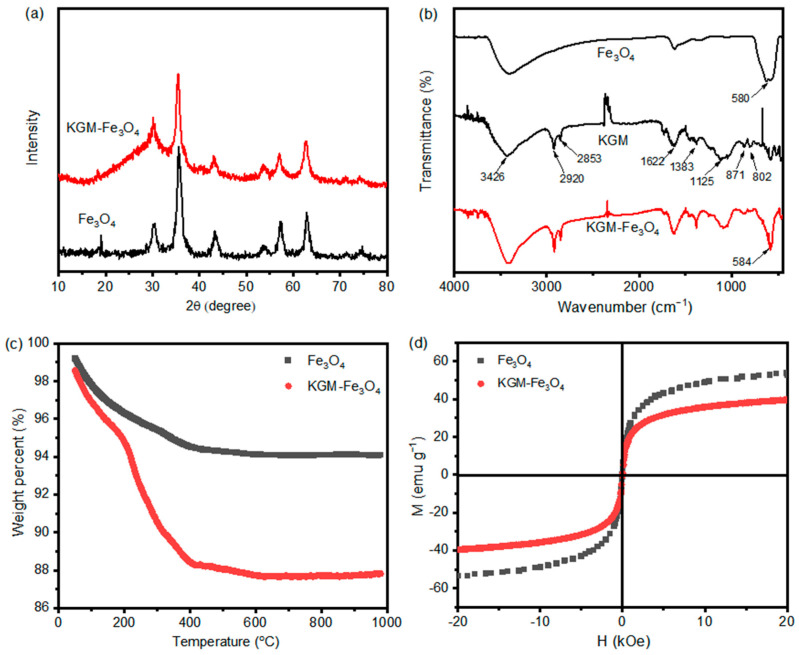
(**a**) XRD patterns of Fe_3_O_4_ and KGM-Fe_3_O_4_ NPs, (**b**) FTIR spectra of Fe_3_O_4_, KGM and KGM-Fe_3_O_4_ NPs, (**c**) TGA thermo grams of Fe_3_O_4_ and KGM-Fe_3_O_4_ NPs, and (**d**) magnetization cures of Fe_3_O_4_ and KGM-Fe_3_O_4_ NPs.

Figure 4a displays the XRD patterns of pure Fe_3_O_4_ and KGM-Fe_3_O_4_ NPs. In Figure 4a, the XRD patterns of pure Fe_3_O_4_ and KGM-Fe_3_O_4_ NPs are almost identical, The characteristic peaks of pure magnetite reflections (JCPDS card no. 75-1610) at 30.1°, 35.4°, 43.1°, 53.4°, 57.0° and 62.6° appeared for both magnetic nanoparticles, corresponding to the crystal planes of (220), (311), (400), (422), (511) and (440), respectively, with high intensity diffraction peaks and no other spurious peaks. It shows that the KGM-based magnetic nanoparticles prepared via one-step reaction precipitation have a cubic phase, and their crystalline phase composition was not changed by the addition of KGM.

Figure 4b shows the FTIR spectra of the pure Fe_3_O_4_, konjac glucomannan and KGM-Fe_3_O_4_ NPs. For pure konjac glucomannan, the broad peak at 3426 cm^−1^ was attributed to the stretching vibration peak of hydroxyl group, and the absorption peaks at 2920 and 2853 cm^−1^ correspond to the symmetric and asymmetric stretching vibration peaks of -CH_2_ on the polymer chain, respectively. The absorption peak at 1622 cm^−1^ is the -OH bending vibration peak of water, the peak at 1383 cm^−1^ is the bending vibration peak of -CH, and the peaks at 1125 and 1039 cm^−1^ belong to the C-O stretching vibration peak and the C-O stretching vibration peak of the C-O-C intracyclic ether on the konjac dextran chain, respectively. The peaks at 800 and 866 cm^−1^ are the characteristic peaks of β-D glucopyranoside. The IR spectra of KGM-Fe_3_O_4_ NPs showed absorption peaks at 3426, 2920, 2853, 1622, 866 and 800 cm^−1^, indicating the presence of konjac dextran on the composites produced [23,28]. In addition, an absorption peak at 580 cm^−1^ corresponding to Fe-O in Fe_3_O_4_ appeared in the spectrum of KGM-Fe_3_O_4_ NPs, indicating the successful preparation of konjac dextran-modified Fe_3_O_4_ nanoparticles.

The TGA analysis of the pure Fe_3_O_4_ and KGM-Fe_3_O_4_ NPs are displayed in Figure 4c. As shown, the weight loss of Fe_3_O_4_ nanoparticles in the temperature range of 0–200 °C was about 3.7% due to the evaporation of residual water in the sample [29], while the weight loss in the range of 200–1000 °C was about 2.23%, which was caused by the phase change weight loss from Fe_3_O_4_ to FeO [30]. The KGM-Fe_3_O_4_ NPs had a weight loss of 5.24% under 200 °C as a result of dehydration. The weight change in the temperature range of 200–1000 °C was mainly due to the degradation of konjac glucomannan [31]. The results indicate that konjac glucomannan was indeed present in the nanoparticles prepared in this experiment, and the content of KGM in the prepared KGM-Fe_3_O_4_ NPs was 6.94%.

The recoverability of KGM-based Fe_3_O_4_ nanoparticles makes it reusable and regenerative in the treatment of heavy metals in wastewater. Figure 4d shows the magnetic hysteresis loop of the as-prepared Fe_3_O_4_ and KGM-Fe_3_O_4_ at room temperature. The coercivity and remanence of Fe_3_O_4_ and KGM-Fe_3_O_4_ NPs were almost unmeasurable, indicating that the prepared samples were all superparamagnetic at room temperature. The saturation magnetization (*M_s_*) of KGM-Fe_3_O_4_ NPs was 39.5 emu·g^−1^, which was slightly reduced compared to that of pure Fe_3_O_4_ (52.7 emu·g^−1^). This was mainly due to the presence of a large amount of non-magnetic konjac dextran in the KGM-Fe_3_O_4_ NPs, since the nanoparticles with up to 16.3 emu·g^−1^ *M_s_* can be separated in the presence of an applied magnetic field [32]. This indicates that the prepared KGM-based Fe_3_O_4_ nanoparticles can achieve rapid solid–liquid separation under the action of an applied magnetic field.

### 2.2. Removal of Cr(VI) Ions by KGM-Based Fe_3_O_4_ Adsorbent

In the adsorption process, the pH value of the solution is an important factor affecting the adsorption capacity of the adsorbent on heavy metal ions, so it is necessary to study the adsorption effect of the adsorbent on heavy metal ions at different pH conditions. The pH of the actual effluent affects the form of Cr(VI) in aqueous solution. At pH > 6.0, Cr(VI) exists mainly as CrO_4_^2−^; in strongly acidic solutions (pH < 1.0), Cr(VI) is mainly in the form of H_2_CrO_4_; at 1.0 < pH < 6.0, the main form of Cr(VI) present is HCrO_4_^−^ [33,34].

**Figure 5:**
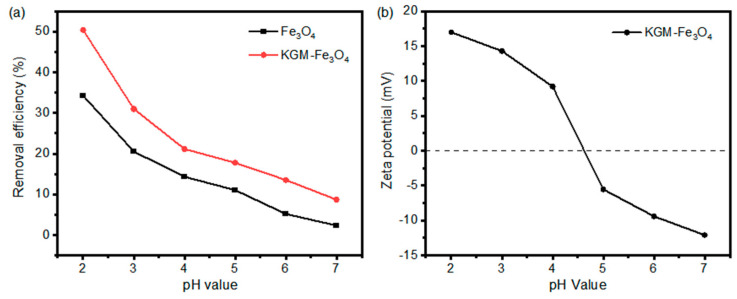
(**a**) Effect of initial pH on the removal of Cr(VI) by KGM-based Fe_3_O_4_ NPs and (**b**) zeta potentials of KGM-Fe_3_O_4_ NPs as a function of pH.

The effects of pH on the removal performance of Cr(VI) by the as-prepared Fe_3_O_4_ and KGM-Fe_3_O_4_ NPs is shown in Figure 5a. It can be seen that the removal efficiencies of the both materials on Cr(VI) decreased gradually with an increase in pH. At a pH of 2.0, the removal efficiencies of Fe_3_O_4_ and KGM-Fe_3_O_4_ NPs on Cr(VI) were 34.28% and 50.42%, respectively, while when the pH was increased to 7.0, the removal efficiencies decreased to 2.23% and 8.70%, respectively. This is mainly related to the zeta potentials of KGM-Fe_3_O_4_ NPs under varying pHs in a solution (as shown in Figure 5b). With the increase in pH, the surface charge of KGM-Fe_3_O_4_ NPs gradually changed from positive to negative with a zero point charge (pHzpc) value of 4.7. That is to say, at a lower pH value (pH < 4.7), the electrostatic attraction between negatively charged HCrO_4_^-^ and KGM-Fe_3_O_4_ NPs resulted in more adsorption of Cr(VI). In addition, comparing the removal efficiencies of the both materials at any pH value, it was found that the KGM-Fe_3_O_4_ NPs had a better adsorption effect than the unmodified Fe_3_O_4_ nanoparticles did, indicating that the modification of Fe_3_O_4_ with konjac dextran had a facilitating effect on the adsorption of Cr(VI). This was mainly due to the presence of a large number of hydroxyl groups on the surface of konjac dextran, and the hydroxyl groups had a good adsorption capacity for Cr(VI).

Although the adsorption of Cr(VI) was better at pH 2.0, Fe_3_O_4_ was partially dissolved at a lower pH. To avoid secondary contamination, pH 4.0 was chosen for the subsequent experiments on balance.

**Figure 6:**
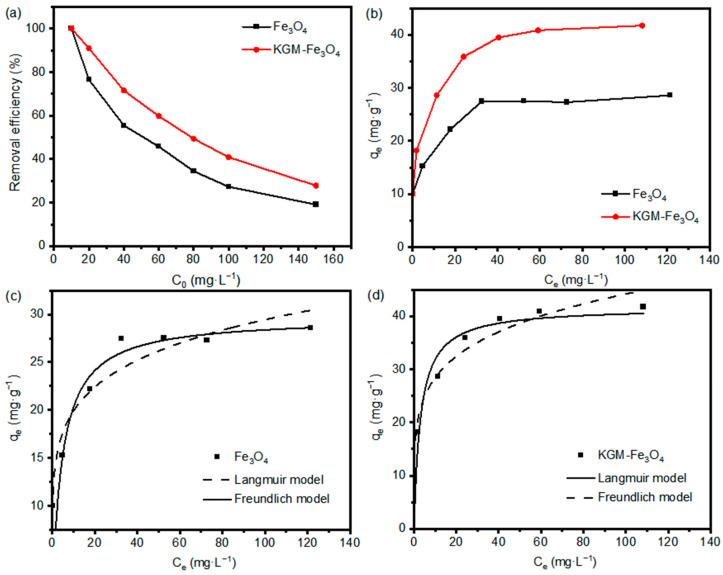
Effect of initial concentration on adsorption of Cr(VI) by KGM-Fe_3_O_4_ NPs: (**a**) removal efficiency, (**b**) adsorption capacity, and non-linear isotherm fitting curves of (**c**) Fe_3_O_4_ and (**d**) KGM-Fe_3_O_4_ NPs.

In order to investigate the adsorption performance of KGM-based Fe_3_O_4_ nanoparticles on Cr(VI), Cr(VI) solutions with different initial concentrations at pH 4.0 were used to investigate the relationship between the adsorption rate and the initial concentration of Cr(VI), and isotherm adsorption models were fitted based on the measured results. As shown in Figure 6a, the Cr(VI) removal efficiency of the samples decreased gradually as the initial concentration increased. When the initial concentration was at a maximum, the Cr(VI) removal efficiencies of the as-prepared Fe_3_O_4_ and KGM-Fe_3_O_4_ NPs were 19.1% and 27.8%, respectively. This is mainly because when the Cr(VI) concentration increases, more Cr(VI) ions in the solution compete with each other to bind to the nanoparticles active sites leading to a decrease in the removal rate. Compared to the unmodified Fe_3_O_4_, KGM-based Fe_3_O_4_ nanoparticles with more active sites resulted in higher removal rates.

Figure 6b shows the isotherms of Cr(VI) adsorption by KGM-based Fe_3_O_4_ nanoparticles. As shown, the adsorption capacity (*q_e_*) rose first, with the gradual increase in the equilibrium concentration (*C_e_*), and then, it tended to level off. In order to study the adsorption mechanism of KGM-based Fe_3_O_4_ nanoparticles on heavy metal ions, the Langmuir and Freundlich adsorption isotherm models were chosen to fit and analyze the experimental data. The non-linear form of two adsorption isotherm model expressions are shown below [35]:

The non-linear form of Langmuir model: $q_{e}=\frac{q_{m}K_{L}C_{e}}{1+K_{L}C_{e}}$.

The non-linear form of Freundlich model: $q_{e}=K_{F}C_{e}^{1/n}$.

Of which, *C_e_* is the concentration of Cr(VI) remaining in the solution at adsorption equilibrium (mg·L^−1^), *q_e_* is the adsorption capacity of the adsorbent at equilibrium (mg·g^−1^), *q_m_* is the maximum adsorption capacity of the adsorbent (mg·g^−1^), *K_L_* is the Langmuir adsorption equilibrium constant (L·mg^−1^), *K_F_* is a constant related to the adsorption capacity, and *n* represents the Freundlich constant.

Figure 6c,d show the non-linear fits of the Langmuir and Freundlich isotherms for the adsorption of Cr(VI) by KGM-based Fe_3_O_4_ nanoparticles. The relevant parameters of the adsorption isotherms are listed in Table 1. As can be seen from Table 1, the linear correlation coefficient *R*^2^ was higher for the adsorption data of the both samples on Cr(VI) using the Langmuir model than that fitted using the Freundlich model, indicating that the Langmuir model is more suitable for describing the adsorption process of KGM-based Fe_3_O_4_ nanoparticles on Cr(VI). That is to say, KGM-based Fe_3_O_4_ nanoparticles during Cr(VI) adsorption belonged to a monolayer adsorption. The maximum adsorption capacities of the both samples for Cr(VI) calculated using the Langmuir model were 29.70 and 41.67 mg·g^−1^, respectively, which were not significantly different from the maximum adsorption capacities of 29.00 and 42.00 mg·g^−1^ measured in the adsorption experiments.

Table 2 shows the comparison of the adsorption capacity of the KGM-Fe_3_O_4_ NP adsorbents with the adsorbents reported in the literature for Cr(VI). The results showed that the adsorption capacity of KGM-Fe_3_O_4_ NPs for Cr(VI) was not the largest among the related adsorbents reported in the literature. However, the adsorbents used in this study were synthesized using a one-step reaction precipitation method, which is a simple and controllable preparation process.

**Figure 7:**
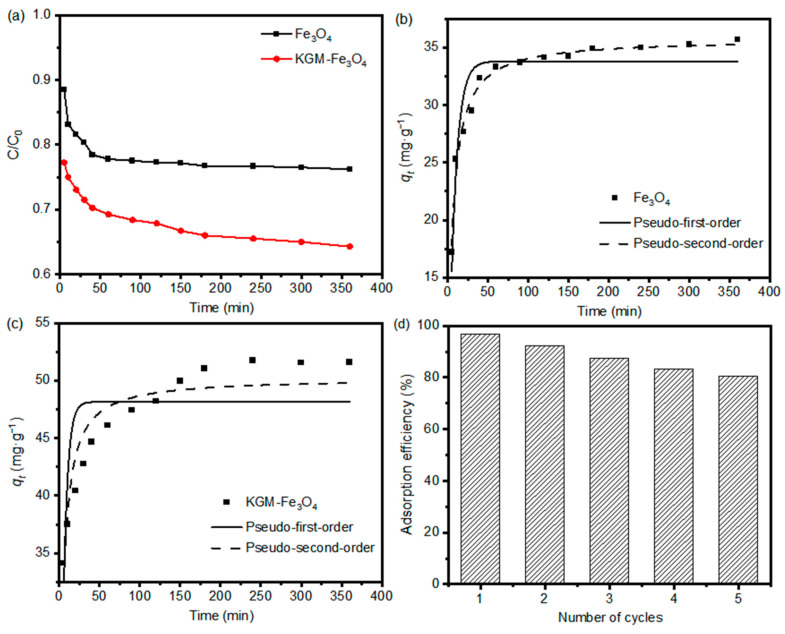
(**a**) Adsorption rate of Cr(VI) (initial concentration of 150 mg·L^−1^) onto the as-prepared Fe_3_O_4_ and KGM-Fe_3_O_4_ NPs, and non-linear kinetic fitting curves of (**b**) Fe_3_O_4_ and (**c**) KGM-Fe_3_O_4_ NPs. (**d**) Reusability of KGM-Fe_3_O_4_ NPs toward Cr(VI) in the adsorption–desorption cycles (dose: 1 g·L^−1^; initial concentration: Cr(VI) 150 mg·L^−1^ at pH 4.0; contact time: adsorption 12 h, desorption 12 h). (**b**) Adsorption capacity.

Moreover, adsorption kinetics is one of the most important features that determine the efficiency of the adsorption process. In this work, the uptake rate of Cr(VI) during adsorption was investigated to characterize the adsorption kinetics. The adsorption rates of the as-prepared nanoparticles for Cr(VI) are shown in Figure 7a. Apparently, due to the strong metal chelating ability of KGM chain, the KGM-Fe_3_O_4_ NPs showed a faster adsorption rate of Cr(VI) compared to that of the pure Fe_3_O_4_ NPs. In other words, KGM plays an important role in improving the adsorption efficiency of the adsorbent on Cr(VI). To further investigate the kinetic properties of Cr(VI) adsorption by KGM-Fe_3_O_4_ NPs, the measured data were non-linearly fitted with pseudo-first-order and pseudo-second-order model equations [35].

Non-linear form of pseudo-first-order kinetic model: $q_{t}=q_{e}(1-e^{-k_{1}t})$.

Non-linear form of pseudo-second-order kinetic model: $q_{t}=\frac{q_{e}^{2}k_{2}t}{1+q_{e}k_{2}t}$.

Where *q_t_* and *q_e_* are the adsorption capacity of the nanoparticles at time *t* and at equilibrium (mg·g^−1^), respectively, *k*_1_ is the first-order kinetic constant (min^−1^), and *k*_2_ is the secondary kinetic constant (mg·g^−1^·min^−1^).

Figure 7b,c shows the non-linear fits of the above kinetics models for the adsorption of Cr(VI) by KGM-based Fe_3_O_4_ nanoparticles, and Table 3 shows the relevant parameters of the models. It can be seen that the linear correlation coefficients *R*^2^ for the pseudo-second-order model were higher than those for the pseudo-first-order model. This indicates that the pseudo-second-order model was more suitable to describe the adsorption process of Cr(VI) by the Fe_3_O_4_ system, i.e., the chemisorption process is the controlling step of this adsorption reaction rate and the adsorbent surface functional group species has a great influence on the adsorption behavior.

In order to assess the reusability of the adsorbent, five adsorption–desorption cycles were carried out using the same adsorbent. Figure 7d shows the relative percentage change in the adsorption capacity of KGM-Fe_3_O_4_ NPs for Cr(VI) during the cycling experiments. As shown, the adsorption capacity of the adsorbent remained around 80% after five cycles. Therefore, the proposed adsorbent has a good regeneration capacity and can be used repeatedly.

## 3. Materials and Methods

### 3.1. Chemical Reagents

Konjac glucomannan concentrate powder was provided by the Konjac Research Centre of Southwest University in Chongqing with 99% konjac content. The other chemical reagents (FeCl_3_·6H_2_O, FeCl_2_·4H_2_O, NaOH and K_2_Cr_2_O_7_) were analytically pure and purchased from Tianjin Guangfu Fine Chemical Research Institute, which were used without further purification. All working solutions were prepared in deionized water with a resistivity of 18.2 MΩ·cm, obtained using a GWA-UN to Pure & Ultrapure water purification system (Purkinje General).

### 3.2. Preparation of KGM-Fe_3_O_4_ NPs

**Figure 8:**
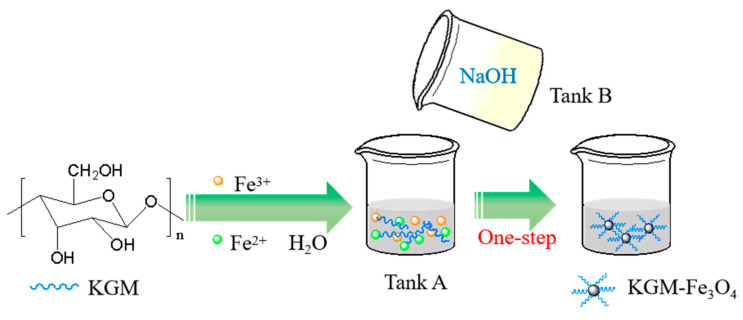
The schematic of the preparation of KGM-Fe_3_O_4_ NPs.

KGM-based magnetic nanoparticles (KGM-Fe_3_O_4_ NPs) were prepared through the one-step reactive precipitation between Fe^3+^, Fe^2+^ ions and NaOH using a traditional stirred tank reactor. In a typical experiment, as shown in Figure 8, FeCl_3_·6H_2_O (2.703 g), FeCl_2_·4H_2_O (1.09 g), deionized water (50 mL) and a certain mass of KGM were mixed in tank A. NaOH (2.4 g) and deionized water (50 mL) were mixed in tank B. The solution in tank B was added to tank A dropwise at 80 °C with vigorous stirring. After that, vigorous stirring was continued for 30 min. Finally, the resulting precipitation was magnetically separated, and then washed with water and ethanol to neutralize it. After freeze-drying, the final products (KGM-Fe_3_O_4_ NPs) were obtained after lyophilized for 8 h. The magnetic Fe_3_O_4_ nanoparticles without konjac glucomannan were obtained under the same experimental conditions.

### 3.3. Batch Adsorption Experiments

All the following adsorption experiments were undertaken in a 250 mL conical flask with stopper containing 50 mL of K_2_Cr_2_O_7_ aqueous solution, in which 1 g·L^−1^ of the as-prepared adsorbent was added. Then, the solution in the conical flask was placed in a thermostatic shaker and shaken at 100 revolutions per minute for a period of time until adsorption equilibrium was reached. The adsorbent was separated from the solution via magnetic separation, and the concentration of Cr(VI) in supernatant liquid was determined via HPLC (U3000, Thermo Fisher Scientific, Waltham, MA, USA) at 350 nm using NH_4_NO_3_ as the mobile phase. The adsorption capacity of the adsorbent for Cr(VI) was calculated as follows:

$$q_{e}=\frac{\left(C_{0}-C_{e}\right)V}{m}$$

Here, *C*_0_ and *C_e_* represent the initial and equilibrium concentrations of Cr(VI) (mg·L^−1^), respectively. *q_e_* denotes the adsorption capacity at equilibrium (mg·g^−1^). *V* is the volume of the Cr(VI) solution (L), and m is the weight of adsorbent (g).

In the course of this study, the effect of pH on the removal of Cr(VI) was investigated at Cr(VI) concentrations up to 150 mg·L^−1^ and pH values ranging from 2.0 to 7.0. The effect of initial concentration on the removal of Cr(VI) was investigated at pH 4.0 with initial Cr(VI) concentrations ranging from 10 to 150 mg·L^−1^. In order to study the effect of adsorption time, the initial Cr(VI) concentration was 150 mg·L^−1^ at pH 4.0, and the dose was 1 g·L^−1^. The adsorbent was then collected at the desired time interval and immediately separated with a magnet to determine the remaining Cr(VI) concentration.

### 3.4. Desorption Test

For the reuse studies, the adsorbents, which had been loaded with Cr(VI) ions, were collected from the adsorption experiments. Then, 5 mM NaOH solution was used as an eluent and mixed with the metal ion-loaded adsorbents. After 12 h, the previously mentioned adsorbents were separated from the eluent with the magnet to obtain the regenerative adsorbents, which were used for the next adsorption measurement. And the released Cr(VI) concentration was analyzed. In order to study the reusability of the adsorbents, five adsorption–desorption cycles were repeated using the same adsorbent. In this study, the adsorption efficiency of the proposed adsorbent was used to assess the reusability. The adsorption efficiency (*E_i_*) of the reusable adsorbent was derived from the following equation:

$$E_{i}=\frac{q_{e,i}}{q_{e,0}}\times 100\%$$

In which, *E_i_* is the adsorption efficiency of the reusable adsorbent after *i* adsorption–desorption cycles (1 ≤ *i* ≤ 5). *q_e,i_* denotes the adsorption capacity of the reusable adsorbent at equilibrium after *i* adsorption–desorption cycles (1 ≤ *i* ≤ 5). *q_e,_*_0_ represents the original equilibrium adsorption capacity of the adsorbent.

All the adsorption and desorption experiments were performed at room temperature (298 ± 2 K), and all experimental data were averaged from triplicate determinations.

### 3.5. Characterization

The morphology was determined using transmission electron microscope (TEM) images (FEI Talos F200x, Thermo Fisher Scientific, Waltham, MA, USA). N_2_ sorption analysis was used to measure the BET-specific surface area of the nanoparticles using an Autosorb iQ-MP (Quantachrome instrument, Boynton Beach, FL, USA). X-ray diffraction (XRD) patterns were obtained in the 2θ range from 10° to 80° with Cu Kα1 radiation (1.5418 Å) using an RIGAKU, D/max-25 X-ray diffractometer at a scanning speed of 5°/min (Rigaku Corporation, Akishima City, Tokyo, Japan). Fourier-transform infrared spectroscopy was applied to the infrared spectra (FTIR, Spectrum Two, Perkin-Elmer, Waltham, MA, USA). Thermogravimetric analysis (TGA) was carried out under a nitrogen atmosphere flowing at a heating rate 10 °C/min using a Rigaku, Thermo Plus EVO TG. Magnetic properties in the range from −20,000 to 20,000 Oe were measured at room temperature with a Vibrating Sample Magnetometer Model 7407 (VSM, Lakeshore Instrument Plant, Columbus, OH, USA). The concentration of Cr(VI) was determined at 350 nm using HPLC (U3000, Thermo Fisher Scientific, Waltham, MA, USA) with NH_4_NO_3_ as the mobile phase.

## 4. Conclusions

In summary, magnetic KGM-based Fe_3_O_4_ nanoparticles (KGM-Fe_3_O_4_ NPs) were prepared via a simple one-step reactive precipitation method. The as-prepared KGM-Fe_3_O_4_ NPs are quasi-spherical with an average diameter of 7.0 nm and saturation magnetization of 39.5 emu·g^−1^. The KGM-Fe_3_O_4_ NPs were applied to remove Cr(VI) from an aqueous solution, and its adsorption isotherm could be described well using the Langmuir isotherm model, indicating that the Cr(VI) ions onto the KGM-Fe_3_O_4_ NPs were monolayer adsorption. The maximum adsorption capacity was 41.67 mg·g^−1^. The adsorption rate of Cr(VI) on the KGM-Fe_3_O_4_ NPs was much higher than that on the pure Fe_3_O_4_ NPs due to the presence of a large number of hydroxyl functional groups on KGM in the KGM-Fe_3_O_4_ NPs. In addition, the adsorption capacity of the KGM-Fe_3_O_4_ NPs for Cr(VI) remained above 80% of the original adsorption capacity after the fifth cycle, which indicated that the KGM-Fe_3_O_4_ NPs adsorbent had remarkable reusability. Based on this review, a reusable magnetic KGM-Fe_3_O_4_ NP adsorbent was prepared through the one-step reactive precipitation method, which is expected to be used for the removal of toxic metal ions from wastewater.

## Figures and Tables

**Table 1:** Isotherm model parameters for Cr(VI) using Fe_3_O_4_ and KGM-Fe_3_O_4_ NPs.

Adsorbent Samples	Langmuir			Freundlich		
	*q_m,cal_* (mg·g^−1^)	*K*_L_ (L·mg^−1^)	*R* ^2^	*K_F_*	*n*	*R* ^2^
Fe_3_O_4_	29.70	0.2164	0.9313	13.47	5.882	0.8241
KGM-Fe_3_O_4_	41.67	0.3234	0.9585	18.26	5.211	0.8624

**Table 2:** Comparison of adsorption capacity of KGM-Fe_3_O_4_ NPs for Cr(VI) with previously reported adsorbents.

Adsorbents	Adsorption Capacity (mg·g^−1^)	Ref.
NZVI/GNS	21.72	[36]
Fe_3_O_4_/bacterial cellulose nanocomposite	25	[37]
Chitosan/PVA/zeolite nanofiber	8.84	[38]
Montmorillonite-supported magnetite nanoparticles	15.3	[4]
polyaniline coating on carbon fiber	18.2	[39]
Modified jacobsite (MnFe_2_O_4_) nanoparticles	31.6	[40]
mesoporous magnetic carbon nanocomposite	3.74	[41]
Functionalized Mesoporous Materials A-10-PG	11.5	[42]
sulfurized nanoscale zerovalent iron (S-nZVI) supported by oyster shell (OS) powder (S-nZVI@OS)	164.7	[43]
KGM-Fe_3_O_4_ NPs	41.67	This work

**Table 3:** Kinetic model parameters for adsorption of Cr(VI) by the as-prepared adsorbents.

Adsorbents	Pseudo-First-Order Kinetics			Pseudo-Second-Order Kinetics		
	*q*_e,cal_ (mg·g^−1^)	*k*_1_ (min^−1^)	*R* ^2^	*q*_e,cal_ (mg·g^−1^)	*k*_2_ (mg·g^−1^·min^−1^)	*R* ^2^
Fe_3_O_4_	33.80	0.1232	0.8900	35.74	5.540 × 10^−3^	0.9837
KGM-Fe_3_O_4_	48.15	0.1912	0.6549	50.23	6.240 × 10^−3^	0.9044

## Data Availability

Not applicable.

## Sample Availability

Samples of the compounds are available from the authors.
